# Molecular Detection and Phylogenetic Analysis of *Riemerella anatipestifer* in Poultry and Wild Geese in Poland

**DOI:** 10.3390/pathogens12020256

**Published:** 2023-02-05

**Authors:** Anna Sawicka-Durkalec, Grzegorz Tomczyk, Iryna Gerilovych, Olimpia Kursa

**Affiliations:** 1 Department of Poultry Diseases, National Veterinary Research Institute, 24-100 Pulawy, Poland; 2 National Scientific Center “Institute of Experimental and Clinical Veterinary Medicine”, 83 Pushkinska, 61023 Kharkov, Ukraine

**Keywords:** *Riemerella anatipestifer*, wild geese, poultry, phylogenetic analysis

## Abstract

*Riemerella anatipestifer* (RA) is one of the most relevant bacterial pathogens of commercial waterfowl from clinical and economic points of view. Our study aimed to evaluate the prevalence of RA infection in different types of commercial poultry in Poland and verify the potential role of wild geese as vectors of this pathogen. We tested a total of 126 poultry flocks, including geese (N = 20), ducks (N = 42), turkeys (N = 64) and 19 wild geese, including greater white-fronted geese (N = 9), greylag geese (N = 5) and Taiga bean geese (N = 5). Tracheal swabs were examined for RA using a PCR targeting a conserved region of the 16S rRNA gene. Selected PCR products were sequenced to perform the phylogenetic analysis. Among the commercial poultry, the highest RA prevalence was found in flocks of ducks (35.7%) and geese (30.0%), whereas the lowest one was found in turkeys (3.2%). Most tested wild geese (94.7%) were RA positive. The phylogenetic analysis showed relatively low genetic diversity of the sequences analyzed, which gathered in two clusters of the phylogenetic tree, and the minimum nucleotide identity was 98.6%. Our results would support the contention that RA isolates from commercial poultry circulate in wild bird populations but are not transmitted back to poultry.

## 1. Introduction

*Riemerella anatipestifer* (RA), previously known as *Pasteurella anatipestifer* or *Moraxella anatipestifer*, is a Gram-negative, rod-shaped bacterium of the Flavobacteriaceae family and *Riemerella* genus [1]. *Riemerella anatipestifer* affects ducklings, goslings, turkeys, other domestic fowl species and also wild birds. The infection can be transmitted horizontally through the respiratory tract and skin wounds [2], but the vertical transmission of RA is also possible [3,4]. However, the exact mechanism of RA transmission among different bird species is still unknown. Cooper [5] speculated that mosquitoes might be vectors for RA transmission in field outbreaks, but this aspect needs to be confirmed in further studies. Infection with RA can cause acute or chronic disease in birds that manifests with respiratory signs, lameness and central nervous system symptoms. Young birds are most susceptible to acute illness with high mortality and morbidity, but RA can also occur in adult birds, causing only subclinical or asymptomatic disease [6]. The disease can be triggered by deterioration in the welfare of the birds, a common cause of which is their transportation, or a sudden change in environmental conditions [7]. Riemerellosis causes substantial economic losses in those countries where ducks are one of the most important branches of animal farming, such as China and countries in Southeast Asia. In Europe, Poland is the third largest producer—after Hungary and France—of duck and goose meat [8,9]. According to Statistics Poland data, the population of ducks in Poland in 2021 was more than 5.2 million, and that of geese more than 892,000 [10]. It is worth noting that the national poultry flock in Poland has been severely reduced in recent years by highly pathogenic avian influenza [11,12]. Economic losses due to RA infections in waterfowl are of significant concern. Its considerable economic impact is caused by increased mortality, reduced growth rates and raised condemnation rates of carcasses at slaughter. *Riemerella anatipestifer* spreads rapidly to various tissues of infected birds, the rapidity depending on the virulence of the strain. The mortality of ducklings infected with *R. anatipestifer* before the fifth week of life ranges from 5% to 75%, and morbidity can reach 100% [2]. Older waterfowl usually suffer chronic, non-fatal or subclinical diseases. Hess et al. [4] found that in commercial flocks of geese infected with RA, clinical signs could occur at 8–10 days of age, and mortality ranged from 5% to 20% and morbidity from 20% to 30%. Viral and bacterial infections specific to the bird species can enhance the pathogenesis of RA. Rubbenstroth [13] reported that avian metapneumovirus was associated with RA infection in turkeys. Infections with RA in waterfowl are commonly reported in coinfections with duck hepatitis virus, duck plague virus, circoviruses, or *E. coli* [14,15,16]. The impact of RA infections in commercial poultry is well documented, and most studies describe clinical cases of RA infection in birds with severe disease symptoms. However, no epidemiological study of RA has yet been conducted in Poland. This study aimed to gain knowledge about the occurrence of RA infection in different types of commercial poultry in Poland, as well as to verify the potential role of wild geese as vectors of this pathogen.

## 2. Materials and Methods

### 2.1. Sampling and DNA Isolation

The survey was carried out in different regions of Poland between 2020 and 2022, and covered three species of commercial poultry, including 20 flocks of geese, 42 flocks of ducks and 64 flocks of turkeys. The swab sampling of live birds was incorporated into the Avian Influenza Monitoring Program that is carried out by the laboratory of the Department of Poultry Diseases of the Polish National Veterinary Research Institute; thus, no ethical permission was needed. Swabbing was performed by experienced veterinarians. All the sampled birds were between 1 and 15 weeks of age and clinically healthy. Oropharyngeal swab samples were collected from 20 randomly selected birds per flock. DNA was extracted from pooled swab samples (10 swabs per pool) suspended in PBS (Biomed, Lublin, Poland). The extraction was performed by an automated magnetic bead-based extraction technique using an IndiMag 48 s instrument and IndiMag Pathogen Kit (Indical Biosci GmbH, Leipzig, Germany). The extracted DNA was frozen immediately and stored at −20 °C until analysis.

Tracheal swabs were taken from 19 wild geese belonging to three species: the greater white-fronted goose (*Anser albifrons*; N = 9), greylag goose (*Anser anser*; N = 5) and Taiga bean goose (*Anser fabalis*; N = 5), that were hunted between 2019 and 2020 in the Warmińsko-Mazurskie voivodeship in the north-eastern Poland. Sampling wild geese by swabbing was performed in cooperation with local hunting authorities during regular hunts carried out as regulations permitted. Oropharyngeal swab samples were collected by an experienced veterinarian immediately after the birds were shot. No ethical approval was needed as all samples were taken post-mortem from birds shot during regular hunts. The samples were collected using swabs with a commercial transport system (ESwab Collection and Transport System, Copan Diagnostic, Murrieta, CA, USA). The DNA was extracted directly from 200 µL of transport medium that was centrifuged at 20,000× *g* for 60 s. The DNA was extracted manually using a QIAamp DNA Mini Kit (Qiagen, Hilden, Germany) following the manufacturer’s recommendations, frozen immediately and stored at −20 °C for further analysis.

### 2.2. Detection of Riemerella anatipestifer DNA with PCR

A PCR assay was performed with primers designed by Tsai et al. [17], targeting a conserved region of the 16S rRNA gene. The 25 μL PCR reaction mixture for the assay was prepared by adding 12.5 μL of EmeraldAmp Max PCR Master Mix (Takara Bio Europe AB, Göteborg, Sweden), 1 μL (10 mM) of forward and reverse primers for RA, 2 μL of the sample and, finally, nuclease free water to make up the reaction volume. All amplification reactions were performed in a T-Personal thermocycler (Biometra, Göttingen, Germany). Ethidium bromide-stained gel electrophoresis (2% agarose gel in 1× Tris-acetic acid-EDTA buffer) was visualized by UV transillumination.

### 2.3. Sequencing and Phylogenetic Analysis

Sequencing was performed on forty-two PCR products that produced strong bands following gel electrophoresis. The bidirectional sequencing (with the forward and reverse PCR primers) of PCR products was performed by the Sanger method in a commercial laboratory (Genomed, Warsaw, Poland). Chromatograms were analyzed and assembled in FinchTV. Thirty-eight high-quality sequences were used for phylogenetic analysis. Phylogenetic analysis was performed in MEGA 11 using the maximum likelihood method and general time reversible model with 1000 bootstrap replication [18]. Bootstrap values of ≥70 were used in the phylogeny [19]. Other sequences available in the GenBank database were included in the analysis. The sequences used in the phylogenetic analysis were deposited in GenBank with accession nos. OP835928–OP835943 and OP863063–OP863084.

### 2.4. Statistical Analysis

The statistical analysis was performed using R version 4.2.1 [20]. Fisher’s exact test was used to verify the differences in the occurrence of *Riemerella anatipestifer* between species of birds using the *RVAideMemoire* package [21]. Differences were considered significant at *p* < 0.05. The results were plotted using *ggplot2* package version 3.4.0 [22]. The map showing the location of sampled and RA-positive poultry flocks was created using QGIS software version 3.28.0 [23].

## 3. Results

**Figure 1:**
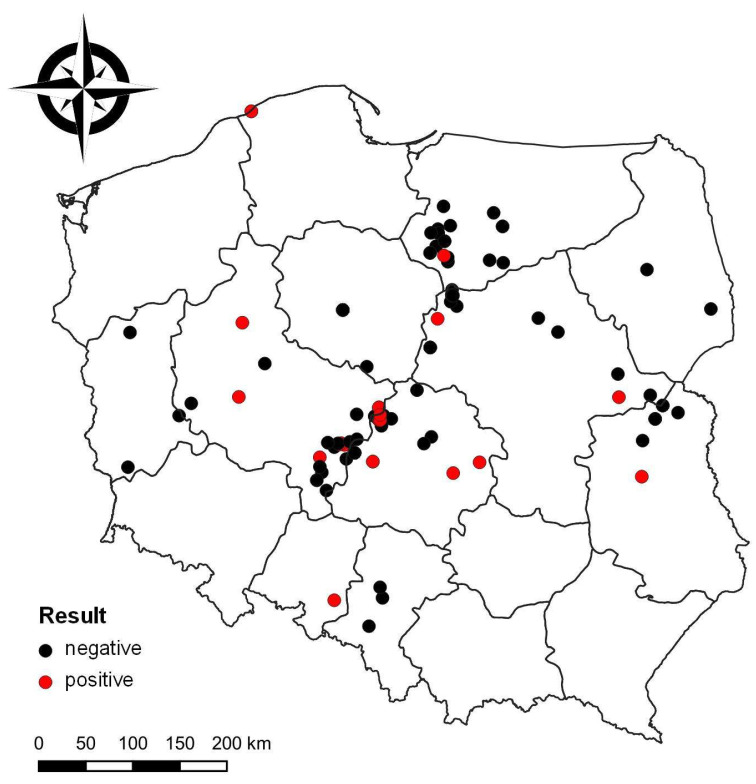
Map showing the locations of sampled commercial poultry flocks. Black dots represent RA-negative flocks and red dots RA-positive ones.

**Figure 2:**
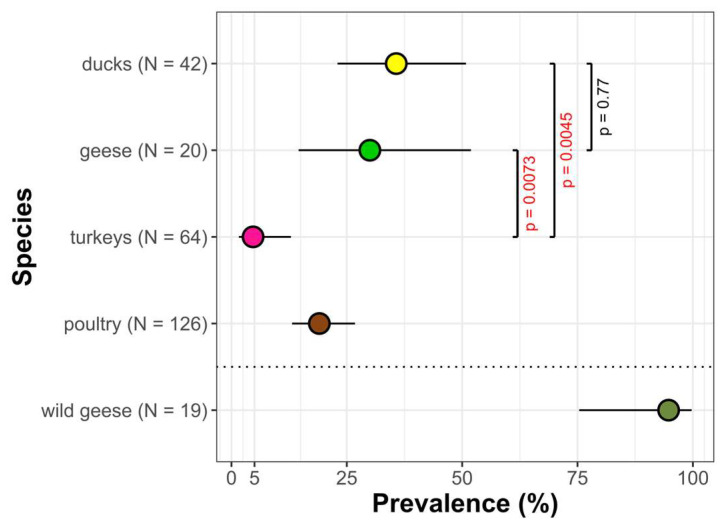
The prevalence of *Riemerella anatipestifer* in different species of commercial poultry and wild geese in Poland. The sample size (n) of poultry species refers to poultry flocks, and the sample size of wild geese refers to the number of individuals. Differences are significant at *p* < 0.05 (Fisher’s exact test).

All the locations where samples were taken and the RA-positivity or -negativity of a tested flock are presented in Figure 1. We tested a total of 126 commercial poultry farms, of which 24 were RA positive (19.0%; 95% CI: 13.1–26.8%). The prevalence of RA was similar in duck (35.7%; 95% CI: 22.9–50.8%) and goose flocks (30.0%; 95% CI: 14.5–51.8%) and the lowest RA prevalence was found in turkeys (3.2%; 95% CI: 0.8–10.8%) (Figure 2). Of the 19 wild geese tested, 18 were RA positive (94.7%, 95% CI: 75.4–99.7%) (Figure 2).

**Figure 3:**
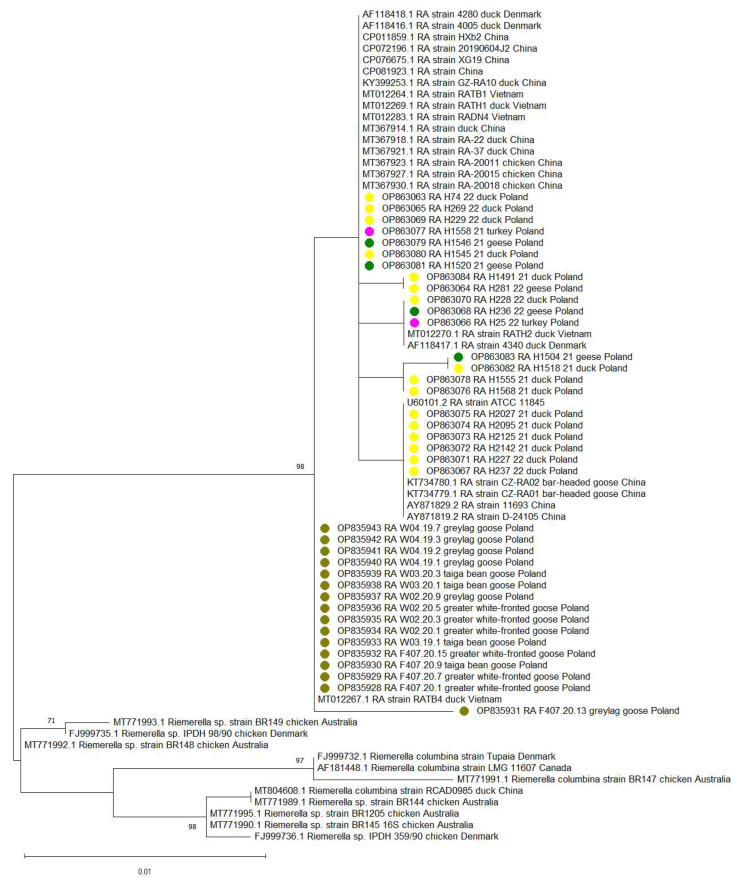
Maximum likelihood phylogenetic tree based on 16S rRNA gene sequences and constructed from a global alignment of 565 bp showing the relationships between 38 *Riemerella anatipestifer* partial sequences obtained in this study and 35 *Riemerella anatipestifer* sequences retrieved from GenBank. Different dot colors represent RA sequences obtained from different bird species in the study (ducks—yellow; commercial geese—green; turkeys—pink; wild geese—olive green).

The phylogenetic analysis showed high similarity among the 38 partial 16S rRNA gene sequences obtained, ranging from 98.6% to 100%. The sequences of Polish RA and selected sequences from GenBank formed two clusters on the phylogenetic tree. The first cluster contained sequences of the 16S rRNA gene of RA obtained from Polish, Chinese, Vietnamese and Danish commercial poultry with a range of similarity of 98.9–100%. This cluster contained six Polish RA sequences obtained from commercial ducklings (GenBank accession nos. OP863067, OP863071–OP863075), which showed identity to the sequence of the ATCC 11845 RA reference strain and two isolates from wild geese from China (Figure 3). Other RA sequences from this study obtained from all three tested species of poultry (GenBank accession nos. OP863063, OP863069, OP863077, OP863079 and OP863080) showed identity with Danish isolates obtained from clinically healthy ducklings (Figure 3). Another sequence obtained from ducks in our study (GenBank accession no. OP863065) showed 99.8% similarity with isolates obtained from Vietnamese commercial ducks with typical signs of RA infection, including septicemia (GenBank accession nos. MT012264, MT012269 and MT012283).

The second cluster included 16 sequences from Polish wild geese and one sequence found in breeding ducks with septicemia from Vietnam (MT012267). The sequence similarity within this cluster ranged from 99.3% to 100%. The sequence obtained from Taiga bean geese (GenBank accession no. OP835938) showed complete identity with the Vietnamese isolate (Figure 3).

## 4. Discussion

*Riemerella anatipestifer* is an important infectious disease that impacts commercial poultry production globally. A large number of case reports of RA infections in poultry are from Asia and mainly concern the duck industry [6,24,25,26,27]. It can be observed, however, that the number of papers describing RA infections in Europe has increased in the last decade. Our studies described RA occurrence in different commercial poultry species in Poland. Infections with RA in commercial geese flocks were also reported in Austria [4] and Hungary [28], Tzora et al. [29] identified an RA infection on a broiler chicken farm located in the southwestern mainland of Greece, and the isolation of RA was also confirmed from a turkey farm located in Croatia [30]. Although Poland is one of the leaders in poultry production in Europe, there is not enough data about the occurrence of RA infections in the country. Our findings demonstrated that the prevalence of RA in duck and goose flocks was higher than in commercial turkey flocks, which corresponds with the findings of studies performed in Hungary by Gyuris et al. [28]. The authors of those studies found a lower prevalence of RA in turkeys than in waterfowl. However, the prevalence of RA in waterfowl found by us was higher than those reported from Hungary. The discrepancy was probably due to the use of different diagnostic methods to determine the occurrence of RA. The prevalence of RA in Polish farmed ducks reported in this study aligns with recent results from Bangladesh, where the authors used a PCR method to detect RA in ducks [31]. However, the Bangladeshi study needs to be interpreted with caution as it has not yet been reviewed.

*Riemerella anatipestifer,* as a globally distributed pathogen, frequently occurs in different orders of wild birds. Outbreaks of the disease have been reported in wild gallinaceous species such as pheasants, guinea fowl, quails and partridges [2]. However, the majority of RA-infected birds reported worldwide were of wild anserid species, including the Canada goose (*Branta canadensis*) [32], snow goose (*Anser caerulescens*), white-fronted goose (*Anser albifrons*), Mandarin duck (*Aix galericulata*), black duck (*Anas rubripes*), wood duck (*Aix sponsa*) [33], black swan (*Cygnus atratus*) [34], mallard (*Anas platyrhynchos*), greater eider duck (*Somateria mollissima*) [35], northern pintail (*Anas acuta*) and spot-billed duck (*Anas poecilorhyncha*) [36]. In our study, 94.7% of wild geese were positive for RA. Similar results were obtained by Cha et al. [36] in South Korea, where a high prevalence of RA (90.0–96.8%) was found in healthy wild ducks, including mallards, northern pintail ducks and spot-billed ducks. A survey carried out in 2015–2016 in western Germany showed high prevalence of RA (70.3%) in Egyptian geese (*Alopochen aegyptiaca*) [37]. Although our study found a high prevalence of RA in wild geese, the phylogenetic analysis of partial 16S rRNA gene sequences did not confirm the relationship between the RA found in wild geese and those found in Polish poultry flocks; it showed the sequences’ similarity to Vietnamese sequences from ducks on breeding farms. Surprisingly, sequences from Polish breeding ducks clustered with sequences from wild geese from China. Our results suggest that RA isolates from commercial poultry can circulate in wild bird populations but are not transmitted back to poultry. Although this requires verification via a larger sample size from wild waterfowl, our results do not provide evidence that wild geese are a vector of RA for stock in the poultry industry. Phylogenetic analysis of 16 Polish sequences showed their close relationship with RA isolates obtained from commercial poultry from China, Denmark and Vietnam (Figure 3). A relatively low genetic diversity was observed for the identified sequences as the nucleotide identity of the sequenced genome fragments was above 98.9%. Nevertheless, our phylogenetic analysis did not reveal a consistent pattern of relationships between sequences obtained from a given poultry species or country of origin. This evidence suggests that closely related RA strains can infect ducks as well as geese and turkeys, the circulation of such strains in commercial poultry flocks thus being a possible source of infection for wild birds. Because of the absence in wild birds of the inhibitory factors operating in poultry farming, such as antimicrobial treatment, RA can survive and circulate in wild birds for a longer period of time.

## 5. Conclusions

In conclusion, our research showed that several RA strains circulate in commercial poultry both at the country level and worldwide. The obtained results highlighted a notable prevalence of RA in domestic waterfowl, namely, ducks and geese. Our findings emphasize the need to implement an appropriate strategy for control and prevention of RA infection in poultry flocks in Poland. The control of RA should be based on the regular monitoring of commercial poultry, using tests with adequate sensitivity and specificity. Given that no commercial vaccine against RA is currently available in Poland, the treatment of riemerellosis in poultry relies mainly on antimicrobial therapy. To further our research, we are planning to evaluate the virulence and antimicrobial susceptibility of RA isolated from Polish commercial poultry.

## Data Availability

The sequences used in the phylogenetic analysis were deposited in GenBank with accession nos. OP835928–OP835943 and OP863063–OP863084.
